# The prevalence, genetic diversity and evolutionary analysis of cachavirus firstly detected in northeastern China

**DOI:** 10.3389/fvets.2023.1233972

**Published:** 2023-09-13

**Authors:** Nuowa Li, Yue Bai, Xin Yan, Zhiyuan Guo, Kongrui Xiang, Zaixing Yang, Haikun Shangguan, Junwei Ge, Lili Zhao

**Affiliations:** ^1^State Key Laboratory for Zoonotic Diseases, Key Laboratory for Zoonosis Research of the Ministry of Education, Institute of Zoonosis, and College of Veterinary Medicine, Jilin University, Changchun, China; ^2^College of Veterinary Medicine, Northeast Agricultural University, Harbin, China; ^3^China Animal Health and Epidemiology Center, Qingdao, China; ^4^Heilongjiang Provincial Key Laboratory of Zoonosis, Harbin, China

**Keywords:** cachavirus, chapparvovirus, phylogenetic analysis, evolution, codon usage

## Abstract

Canine cachavirus is a novel parvovirus belonging to the genus *Chaphamaparvovirus* that was first detected in dogs in the United States. However, our knowledge of the prevalence and genetic characteristics of cachavirus is relatively limited. In this study, 325 canine fecal specimens collected from healthy and diarrheic dogs in northeastern China were screened with PCR. Twenty-two of the 325 (6.8%) samples were positive for cachavirus. The diarrhea samples showed high viral coinfection rates, and we detected coinfections with canine astrovirus (CaAstV) and cachavirus for the first time. A sequence analysis revealed that the Chinese cachavirus strains have point mutations in four consecutive amino acid codons relative to the original American strain. A codon usage analysis of the VP1 gene showed that most preferred codons in cachavirus were A- or T-ending codons, as in traditional canine parvovirus 2. A co-evolutionary analysis showed that cachavirus has undergone cospeciation with its hosts and has been transmitted among different host species. Our findings extend the limited cachavirus sequences available, and provide detailed molecular characterization of the strains in northeastern China. Further epidemiological surveillance is required to determine the significance and evolution of cachavirus.

## 1. Introduction

Cachavirus, which belongs to the species *Chaphamaparvovirus carnivran1*, is a small, icosahedral, nonenveloped virus. It has a single-stranded DNA genome containing two major opening reading frames encoding nonstructural protein 1 (NS1) and the capsid protein (VP1) (1). In recent years, the family *Parvoviridae* has been re-organized by the International Committee on Taxonomy of Viruses, and the new subfamily *Hamaparvovirinae* now includes the genus *Chaphamaparvovirus*, which have been detected in several hosts (2). Chaphamaparvoviruses include bat (*Eidolon helvum*) parvovirus 2 (EHPV2) (3), common vampire bat (*Desmodus rotundus*) parvovirus (4), fruit bat (*Eidolon helvum*) parvovirus 1 (5), etc. Several studies have also detected chaphamaparvoviruses in mammals such as simians (6), Tasmanian devils (7), rat (8, 9), swine (10), and birds, including turkey (11), red-crowned crane (12), chicken (13), and peafowl (14).

Cachavirus, which belongs to the genus *Chaphamaparvovirus*, is a novel canine parvovirus first detected in dogs with diarrhea in the United States in 2019. Cachavirus infections have been associated with both asymptomatic infections and clinical signs such as diarrhea, and the virus tends to establish active infections in already-infected hosts (15). Notably, a recent study reported the detection of two strains of cachavirus in the feces of pet cats, which may have originated in dogs (16). Cachavirus was also found in wild coyotes in 2022, and a study indicated that the virus had been circulating in wild animals for at least 10 years (15, 17). These results confirm that cachavirus has multiple hosts and a capacity for cross-species transmission. Therefore, further research is required. Although cachavirus has been detected in several geographic locations, including the United States, Canada, and some provinces of China (18), our knowledge of the prevalence and genetic characteristics of the virus is relatively limited.

In this study, we collected 325 fecal samples from dogs in four provinces of northeastern China (Inner Mongolia Autonomous Region, Heilongjiang, Liaoning, and Jilin) and looked for cachavirus with PCR. We also examined the rate of cachavirus in mixed infections with other viruses. To examine the prevalence of and risk factors for cachavirus infection, we compared the collection date and location, and the host age of the positive samples. We subjected the detected cachavirus and known chapparvovirus sequences to a phylogenetic analysis, and investigated the codon usage bias in the cachavirus VP1 gene. We also performed a co-evolutionary analysis to investigate the development and transmission of cachavirus and other chaphamaparvoviruses.

## 2. Materials and methods

### 2.1. Sampling

Fecal samples from 325 diarrheic and healthy dogs were collected in the Inner Mongolia Autonomous Region, Heilongjiang, Liaoning, and Jilin Provinces in 2019–2021. The samples were collected from both healthy (*n* = 40) and diarrheic dogs (*n* = 285). Details of all the samples, including the time and place of collection and the host’s age, immune status, and clinical symptoms, were recorded. The fecal samples were stored at −20°C until analysis.

### 2.2. Sequence amplification and analysis

To isolate the viral DNA and RNA, we used the EasyPure® Viral DNA/RNA Kit (TransGen Biotechnology Co., Ltd., Beijing, China). The samples were stored at −80°C (19, 20). Four primer pairs (Table 1) produced by Comate Bioscience (Changchun, China) were used with PCR to amplify the NS1 and VP1 genes of cachavirus from the positive samples (16). The PCR products were purified and sequenced with the Sanger method. All PCRs and genome sequencing were performed with at least three replicates (21).

**Table 1:** Primers used for PCR amplification and sequence analysis.

Virus	Primers	Sequences (5′-3′)	Sense	Amplicon size	Use
Cachavirus	ChPV-1F	TGACTGGTTAGTTCGCTTTC	+	1,099 bp	Amplification
	ChPV-1R	GGTTCTTCCCATACTCCAAT	−		
	ChPV-2F	GCTATTATGATTTAGGAGAACGCTT	+	1,098 bp	Amplification
	ChPV-2R	CTGGTTCGTATCCCGTCGCTA	−		
	ChPV-3F	CTCCTGCACCTCAGTTAGCG	+	935 bp	Amplification
	ChPV-3R	GCCATACAGCCGATCCAC	−		
	ChPV-4F	TGCACAAGATGATCTATACGAA	+	1,294 bp	Amplification
	ChPV-4R	GGATACACAGGCGCCAGTACAGTA	−		
	ChPV-OF	CAACTAGCCGAATGCAGGGA	+	323 bp	Screening
	ChPV-OR	CGATAACATCCCCGGACTGG	−		
CDV	P1	ACAGGATTGCTGAGGACCTAT	+	287 bp	Screening
	P2	CAAGATAACCATGTACGGTGC	−		
CPV	CPV3381-F	CCATGGAAACCAACCATACC	+	717 bp	Screening
	CPV4116-R	AGTTAATTCCTGTTTTACCTCCAA	−		
AstV	AsTVs_625F-1	GTACTATACCRTCTGATTTAATT	+	300 bp	Screening
	AsTVs_626R-1	AGACCAARGTGTCATAGTTCAG	−		
CCoV	CCV1	TCCAGATATGTAATGTTCGG	+	409 bp	Screening
	CCV2	TCTGTTGAGTAATCACCAGCT	−		

### 2.3. Screening for canine coinfecting pathogens

The collected samples were also screened for canine parvovirus 2 (CPV-2) (22), canine coronavirus (CCoV) (23), canine astrovirus (CaAstV) (24), and canine distemper virus (CDV) (25), which were then sequenced with the method described above.

### 2.4. Recombination analysis

To detect any recombination signals in the nucleotide sequences of the cachavirus strains, we used the Recombination Detection Program package 4 (RDP4) to identify recombination events (26). Recombination events were considered significant when detected by at least 4 methods with *p* ≤ 0.01.

### 2.5. Phylogenetic analysis

To analyze the sequences and demonstrate the phylogenetic relationships of cachavirus, we used the DNAStar software (DNASTAR, Madison, WI, United States). We downloaded all the available reference sequences of cachavirus strains and several sequences of feline chaphamaparvovirus from the GenBank database, and aligned them with the sequences obtained in this study using the ClustalW method, MegAlign program of DNAStar (DNAStar, Madison, WI, United States). We used the neighbor-joining method in the MEGA 7.0 software to construct phylogenetic trees based on the nucleotide sequences of the VP1 and NS1 genes, with 1,000 bootstrap replicates to evaluate the statistical support (27).

### 2.6. Codon usage analysis

The frequencies of the basic nucleotides (A%, T%, C%, and G%), nucleotide at the third position of synonymous codons (A3%, T3%, C3%, and G3%), and the GC and AT contents were calculated for the VP1 gene sequence of each cachavirus strain with CodonW version 1.4.2. In order to better show the codon usage preference of Chinese cachavirus strains, we analyzed the strains detected in this study and reference strains from the United States and China. The effective codon number (ENC) and relative synonymous codon usage (RSCU) were also calculated with CodonW (28).

### 2.7. Co-evolutionary analysis

To estimate the virus–host codivergence, we simultaneously analyzed the phylogeny of all available chaphamaparvoviruses, together with their hosts’ phylogenies. The phylogenetic tree of the viruses was constructed based on their complete genomes, and the phylogenetic tree of the host species was based on their mitochondrial genomes. To estimate the relative frequencies of co-evolution and cross-species transmission between chaphamaparvoviruses and their hosts, we performed a co-phylogenetic analysis with the Jane package v4 (29) with 0–1–1-1-1 parameters (30). The host tree was drawn in black and the pathogen tree in blue.

## 3. Results

### 3.1. Prevalence of cachavirus

Among the 325 samples collected in northeastern China, 22 (6.8%) were positive for cachavirus. The positive rates in Heilongjiang, Jilin, Liaoning, and Inner Mongolia were 12.8, 3.8, 1.1, and 2.6%, respectively. The prevalence rate in Heilongjiang Province was obviously high, whereas those in the other three provinces were relatively low. Detailed information about the cachavirus-positive samples is listed in Table 2. The detection rate in the diarrhea samples was 6.3% (18/285), and only one positive dog was dying. The detection rate in healthy samples was 10% (4/40). The positive rate of cachavirus was highest in dogs between 2 and 4 months old (16/22, 72.7%), and the positive samples were concentrated in December to February (18/22, 81.8%), showing a clear seasonal trend. This obvious seasonal tendency was probably attributable to specific seasonal changes in northeastern China. The majority of the positive samples (54.5%, 12/22) were from unvaccinated dogs; 13.6% (3/22) of hosts experienced incomplete vaccination programs, and 31.8% (7/22) completed the vaccination programs.

**Table 2:** The details about the positive samples from which the viruses were detected.

Strain	Location	Sampling month	Age (months)	Sample type	Clinical symptoms	Status of immunity	Other enteric pathogens detected
A2	Harbin	2019.11	2	Stool	Diarrhea	Not immunized	AsTV+CCoV
A7	Harbin	2020.1	2	Stool	Diarrhea	Complete immunity	CCoV
A8	Harbin	2019.11	3	Stool	Asymptomatic	Complete immunity	CCoV
A9	Harbin	2020.1	2	Stool	Diarrhea	Complete immunity	AsTV+CDV + CCoV
A10	Harbin	2020.1	4	Stool	Diarrhea	Not immunized	AsTV+CDV + CCoV
A11	Harbin	2020.1	4	Stool	Diarrhea	Partial immunity	CCoV
B3	Yanji	2020.1	12	Stool	Asymptomatic	Complete immunity	CCoV
B7	Yanji	2020.1	14	Stool	Asymptomatic	Complete immunity	CCoV
D2	Mudanjiang	2019.11	2	Stool	Diarrhea	Complete immunity	CCoV
D4	Mudanjiang	2019.12	3	Stool	Diarrhea	Not immunized	CCoV
D6	Mudanjiang	2020.1	12	Stool	Diarrhea	Complete immunity	AsTV+CDV + CCoV
D7	Mudanjiang	2020.1	3	Stool	Diarrhea	Not immunized	CCoV
D9	Mudanjiang	2020.1	3	Stool	Diarrhea	Not immunized	CCoV
F10	Heihe	2020.4	2	Stool	Diarrhea	Not immunized	CCoV
F17	Heihe	2020.12	4	Stool	Diarrhea	Not immunized	CCoV
F19	Heihe	2020.12	4	Stool	Diarrhea	Not immunized	CCoV
F20	Heihe	2020.12	4	Stool	Diarrhea	Not immunized	CCoV
G2	Qiqihar	2019.12	5	Stool	Diarrhea	Not immunized	CCoV
G15	Qiqihar	2020.2	11	Stool	Diarrhea	Partial immunity	CCoV
G17	Qiqihar	2020.2	4	Stool	Diarrhea	Partial immunity	CCoV
H1	Hulun Buir	2020.1	12	Stool	Diarrhea	Not immunized	CCoV
K46	Shenyang	2020.12	3	Anal swab	Asymptomatic	Not immunized	CCoV

Coinfections of cachavirus with other viruses were detected in all 22 positive samples. The coinfection of cachavirus-positive samples was presented in Supplementary Figure S1. In the detection of this study, 22 cachavirus-positive samples were coinfected with CCoV (22/22, 100%), 18.2% with CaAstV (4/22), and 13.6% with CDV (3/22). Of the coinfected samples, 13.6% (3/22) were simultaneously coinfected with the three intestinal pathogens CCoV, CDV, and CaAstV, and one sample was coinfected with both CCoV and CaAstV. Among the samples with mixed infections of cachavirus and CCoV, four were had no signs of disease, but 14 hosts had clinical signs. All dogs with other complex coinfections had obvious diarrhea. We also observed a dog near death that was infected with cachavirus and multiple viruses.

### 3.2. Genetic characterization

We compared the nucleotide and amino acid mutations in three NS1 and three VP1 sequences of cachaviruses determined in this study and those of 16 reference strains (Supplementary Table 2). The nucleotide identity was 92.5–99.6% and amino acid identity 98.1–100% for the 19 VP1 sequences. A sequence comparison of the NS1 gene revealed nucleotide identities of 92.1–99.8% and amino acid identities of 97.6–100%. No recombination signal was detected in the aligned DNA sequences.

A comparison of the deduced cachavirus VP1 proteins detected the following mutated sites in the three strains in this study: Gln149Leu, Val265Ile, Thr422Pro, Asn427Thr, and Arg449Gly (Table 3). Strains A8 and K46 identified in this study showed unique mutation sites at residues 427 (Asn → Thr) and 449 (Arg → Gly), whereas F10 showed amino acid changes at residues 149 (Gln → Leu) and 422 (Thr → Pro). The VP1 sequences of two cachaviruses identified from cats also showed many different mutant sites. A comparison of the amino acid sequences of the deduced NS1 protein in the cachaviruses identified four major mutant sites: Ser252Cys, Gly253Leu, Gly254Thr, and Tyr255Phe (Table 4). Notably, this continuous amino acid change is common in cachaviruses detected in China, Canada, and Italy in previous studies. Strains CY56 and NWT-W78 showed similar mutant sites in both the NS1 and VP1 sequences, which differed in this way from those of all other available strains.

**Table 3:** Main amino acid mutation sites in VP1 of cachavirus.

Isolate	Substitution of amino acid residues in VP1																			
	22	56	68	130	131	149	265	326	332	340	365	402	411	422	427	437	445	448	449	460
Cachavirus-1A	Y	I	Y	D	F	Q	V	Y	G	Y	Q	D	R	T	N	F	T	S	R	K
Cachavirus-1B	Y	I	Y	D	F	Q	I	Y	G	Y	Q	D	R	T	N	F	T	S	R	K
MT123283	Y	I	Y	D	F	Q	V	N	G	Y	Q	D	R	T	N	L	T	S	R	K
MT123284	Y	I	Y	D	F	Q	I	Y	G	Y	Q	D	G	T	N	F	T	S	K	K
MT123285	Y	I	Y	D	F	Q	V	Y	G	C	Q	D	R	T	N	F	T	S	R	K
MT123286	Y	I	Y	D	F	Q	V	Y	G	Y	Q	D	R	T	N	F	T	S	R	K
MT123287	Y	I	Y	D	F	Q	I	Y	R	Y	Q	D	R	T	N	F	T	S	R	K
MT710947	Y	I	Y	D	F	Q	V	Y	G	Y	Q	D	R	T	N	F	T	S	R	K
MT710948	Y	I	Y	D	F	Q	V	Y	G	Y	Q	D	R	T	N	F	T	S	R	K
OK546100	Y	I	Y	D	F	Q	I	Y	G	Y	Q	D	R	T	N	F	T	S	R	K
OK546101	Y	I	Y	D	F	Q	I	Y	G	Y	Q	D	R	T	N	F	T	S	R	K
OK546102	Y	I	Y	D	F	Q	I	Y	G	Y	Q	D	R	T	N	F	S	S	R	K
OM640108	Y	I	Y	B	F	Q	I	Y	G	Y	Q	D	R	T	N	F	S	T	R	K
OM640109	Y	I	Y	D	F	Q	I	Y	G	Y	Q	D	R	T	N	F	S	S	R	R
MN928790	Y	T	C	D	S	Q	I	Y	G	Y	Q	N	R	T	N	F	T	S	K	K
MN928791	H	I	Y	D	F	Q	I	Y	G	Y	R	D	R	T	N	F	T	S	K	K
A8	Y	I	Y	D	F	Q	I	Y	G	Y	Q	D	R	T	T	F	T	S	G	K
F10	Y	I	Y	D	F	L	I	Y	G	Y	Q	D	R	P	N	F	T	S	R	K
K46	Y	I	Y	D	F	Q	I	Y	G	Y	Q	D	R	T	T	F	T	S	G	K

**Table 4:** Main amino acid mutation sites in NS1 of cachavirus.

Isolate	Substitution of amino acid residues in NS1																
	8	13	49	53	58	62	186	198	228	247	248	252	253	254	255	270	272
Cachavirus-1A	G	T	A	Q	S	P	E	R	O	S	I	S	G	G	Y	O	H
Cachavirus-1B	G	T	A	Q	S	P	E	R	O	S	I	S	G	G	Y	O	H
MT123283	G	T	A	Q	A	P	E	R	O	S	I	C	L	T	F	O	H
MT123284	G	T	A	Q	S	P	E	R	O	S	I	C	L	T	F	R	H
MT123285	G	T	A	R	S	P	E	R	R	S	I	S	V	T	F	O	H
MT123286	G	T	A	Q	S	P	E	R	R	S	I	S	V	T	F	O	H
MT123287	G	T	A	Q	S	P	E	R	O	P	I	S	V	T	F	R	H
MT710947	G	T	A	Q	S	P	E	R	O	S	I	C	L	T	F	O	H
MT710948	G	T	A	Q	S	R	E	R	O	S	I	C	L	T	F	O	H
OK546100	G	T	A	Q	S	P	E	H	O	S	I	C	L	T	F	O	H
OK546101	G	T	A	Q	S	P	E	R	O	S	I	C	L	T	F	O	H
OK546102	S	T	A	Q	S	P	K	R	O	S	I	C	L	T	F	O	X
OM640108	G	T	X	Q	S	P	E	R	O	S	I	C	L	T	F	X	H
OM640109	S	I	A	Q	S	P	E	R	O	S	I	C	L	T	F	O	H
MN928790	G	T	A	Q	S	P	E	R	O	S	I	C	L	T	F	O	H
MN928791	G	T	A	Q	S	P	E	R	O	S	V	S	V	T	F	O	H
A8	G	T	A	Q	S	P	E	R	O	S	I	C	L	T	F	O	H
F10	G	T	A	Q	S	P	E	R	O	S	I	C	L	T	F	O	H
K46	G	T	A	Q	S	P	E	R	O	S	I	C	L	T	F	O	H

### 3.3. Phylogenetic analysis of cachavirus

**Figure 1:**
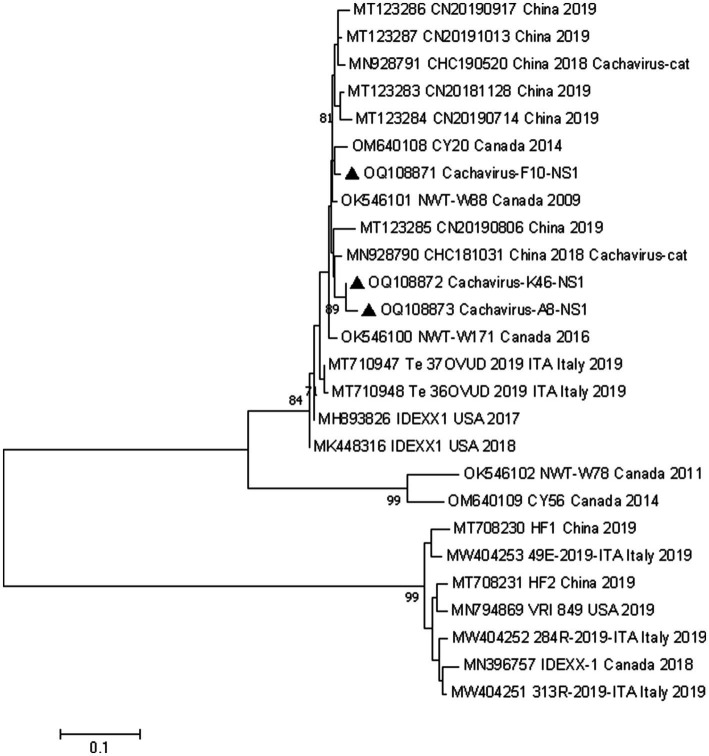
Phylogenetic analysis of cachavirus strains based on the nucleotide sequences of the partial NS1 gene. Bar indicates genetic distance. Triangle represents the cachavirus strains identified in this study.

**Figure 2:**
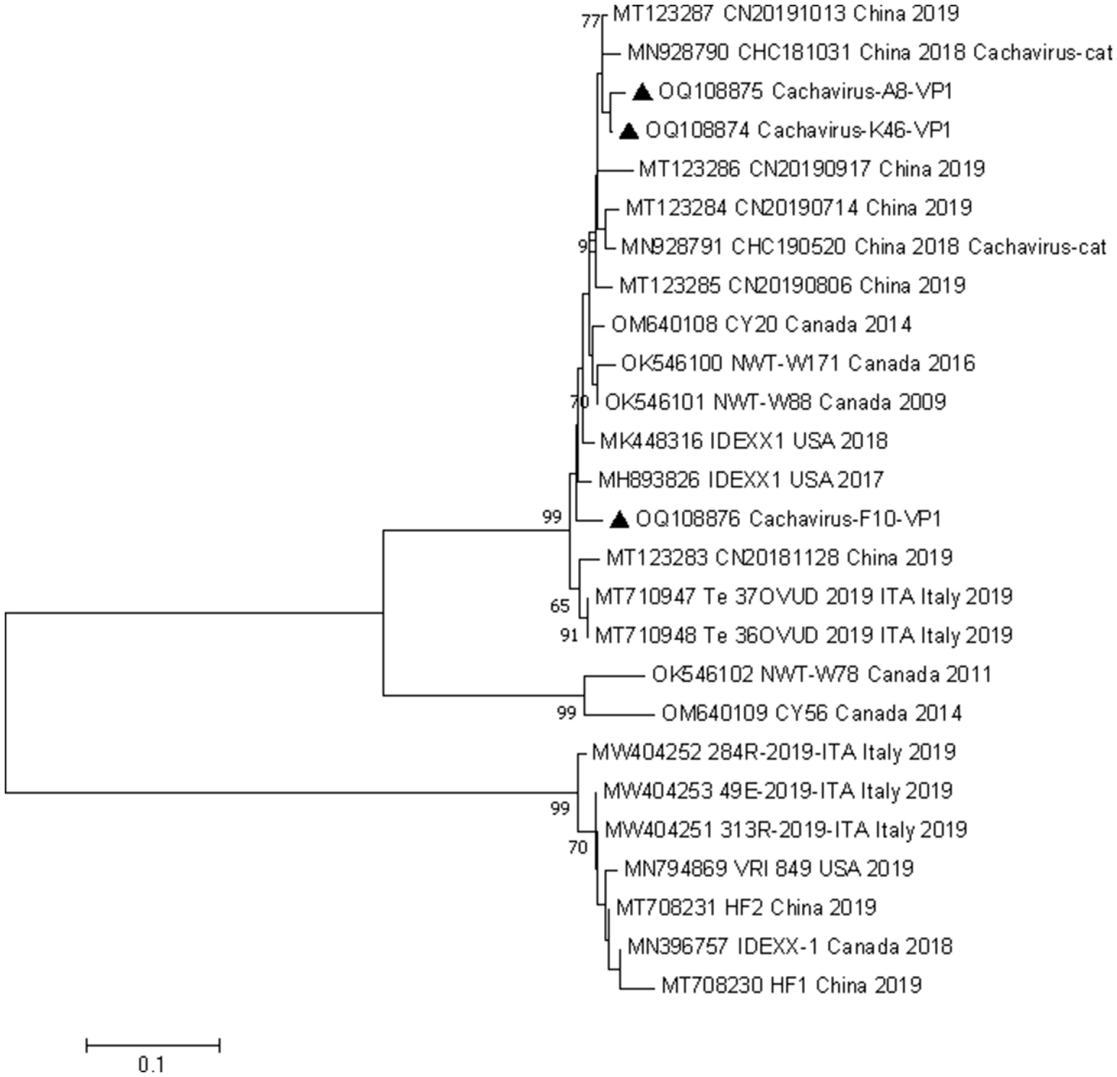
Phylogenetic analysis of cachavirus strains based on the nucleotide sequences of the partial VP1 gene. Bar indicates genetic distance. Triangle represents the cachavirus strains identified in this study.

We conducted phylogenetic analyzes of the NS1 and VP1 genes based on 26 nonrecombinant sequences of cachaviruses and feline chaphamaparvoviruses. As shown in Figure 1 and Figure 2, on these phylogenetic trees, the cachavirus sequences identified in canines formed two clades: one composed of OM640109 and OK546102, identified in coyotes and wolves, respectively, with 100% bootstrap support. All other sequences identified in canines (mainly dogs), plus MN928791 and MN928790, which were from cats, formed a single cluster. The three Chinese strains detected in this study were all in one clade with the reference strains identified in canines, cats, and wild animal. This suggests that cachavirus has evolved as it has spreads.

### 3.4. Codon usage analysis

We analyzed the basic nucleotide composition of the VP1 gene of cachavirus. In 12 cachavirus strains, codons with RSCU >2, including TTT, TTA, TCT, GCA, AGA, and GGA, were overrepresented in VP1 (Supplementary Table 1). This finding indicates that phenylalanine, serine, arginine, alanine, glycine, and leucine are preferentially coded by TTT, TCT, AGA, GCA, GGA, and TTA, respectively, in the cachavirus VP1 gene. The codons TTT (Phe), TCT (Ser), GAA (Glu), TGT (Cys), GCA (Ala), CAT (His), GAT (Asp), and TGG (Trp) were favored in all cachavirus strains.

Only codon GTT (Val) is used preferentially in strains cachavirus-1A, cachavirus-1B, MT123283, MT123284, MT123285, MN928790, F10, and K46, whereas no obvious codon bias was detected in strain MT123286, MT123287, MN928791, or A8. The RSCU values and terminal nucleotide compositions indicated that A- or T-ending codons were strongly favored in the VP1 coding sequences of cachavirus, whereas terminal-G and -C codons were rare. Further analysis showed that most amino acids in the cachavirus VP1 protein are encoded by codons ending in A or T.

To understand the extent to which codon usage deviates from random selection, it is usually necessary to describe it with the ENC value. The ENC value of the cachavirus strains ranged from 39.08 to 41.57, with an average value of 40.56 and a standard deviation (SD) of 0.72, and this high ENC value (> 40) (28) indicates low codon use bias in the cachavirus VP1 gene.

### 3.5. Co-evolutionary analysis of chapparvovirus

**Figure 3:**
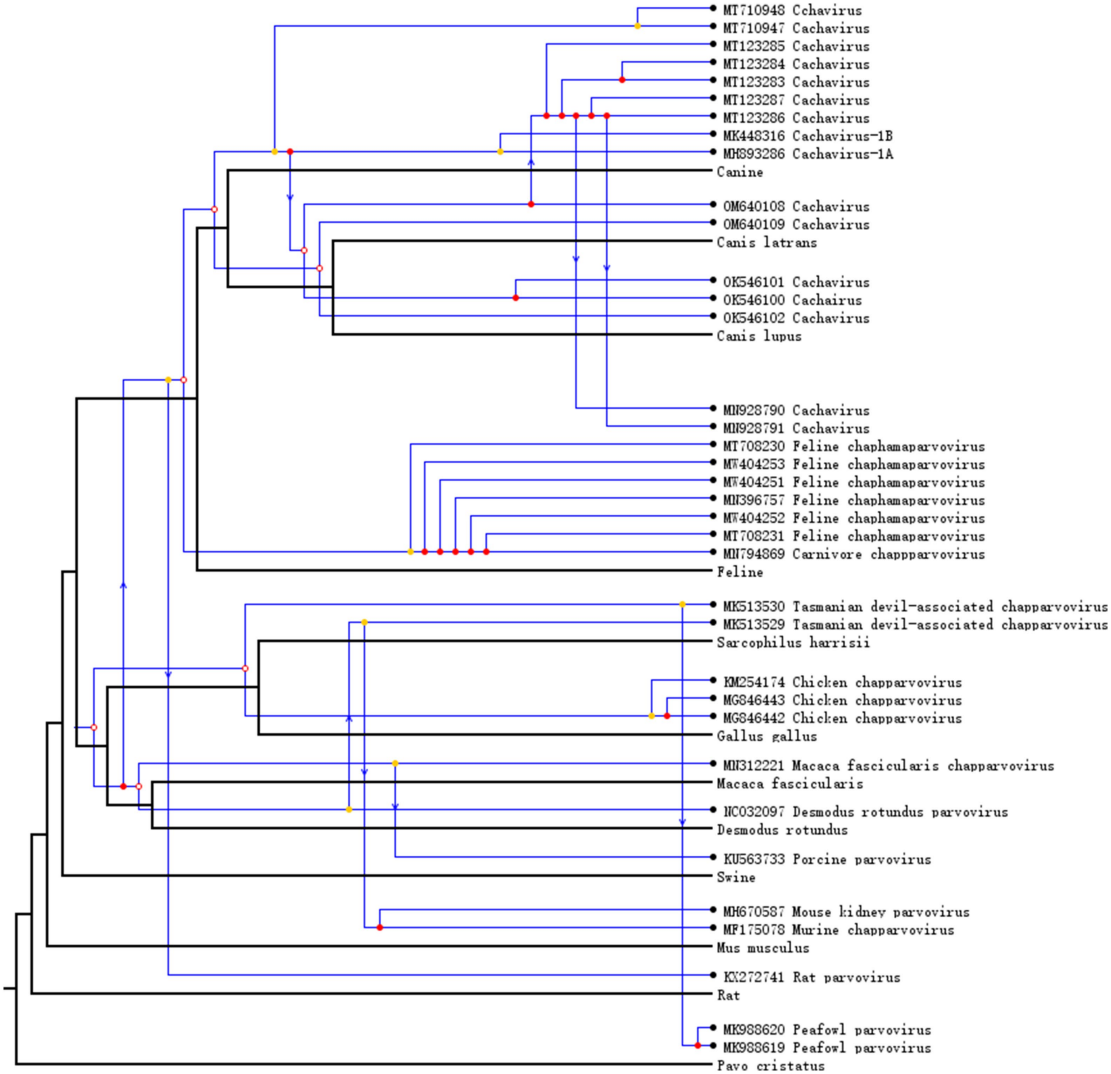
Host–virus co-phylogenetic analysis of Chapparvovirus. Cospeciation, host-switching, and duplication events are labeled with empty circles, arrows, and filled circles, respectively.

To understand the co-evolution and cross-species transmission of chaphamaparvoviruses and their hosts, we compared the tree topologies of viruses and their host. Chaphamaparvoviruses and their hosts (and associated taxa) are connected by lines. As shown in Figure 3, the relative frequencies of the evolutionary events tested were seven codivergence events, 18 for duplication events, and 10 host-switching events. Cachavirus showed significant cospeciation with feline chaphamaparvovirus, and multiple replication events and host-switching events have occurred in canine cachavirus. Cats can be infected by both cachaviruses and feline chaphamaparvovirus. The cachavirus in coyotes showed significant cospeciation with the cachavirus in wolves. When the analysis was extended to the phylogenies of different chaphamaparvoviruses and their hosts, the history of their evolution was explained with more host-jumping and cospeciation events.

## 4. Discussion

At present, cachavirus infections are associated with clinical signs such as diarrhea, and the virus has been detected at limited geographic locations, including the United States and some provinces of China. However, the prevalence and genetic characteristics of the virus are relatively unknown. In the present study, we investigated the prevalence and coinfection rates of cachavirus in the entire northeastern region of China. We also conducted molecular and phylogenetic analyzes to better understand the evolution of cachavirus and to provide new directions for its control.

Cachavirus is highly prevalent in northeastern China, with a large geographic spread. The overall positivity rate in dogs was 6.8%, which is higher than previously reported in China (1.55%) (16) or in two recent studies in Canada [2.6% (15) and 3.3% (17)]. Cachavirus, a recently identified novel canine parvovirus, is spreading around the world and has been detected in the United States, Canada, Italy, and several provinces of China. Its geographic reach has increased our concern about this virus. Our results show that the positivity rate of cachavirus varies with the host’s age, immune status, and clinical symptoms and the collection date and location of the sample in northeastern China. Its preferred host age is clearly young dogs (puppies) and its seasonal preference is winter. Therefore, given the high prevalence of cachavirus, we must pay more attention to puppies, especially in winter.

The pathogenicity of cachavirus requires further study, especially with animal experiments. To date, reports of the pathogenicity of chaphamaparvoviruses in vertebrate hosts have been relatively limited. Mouse kidney parvovirus (MKPV) has been shown to cause a kidney disease called “inclusion body nephropathy” in a population of laboratory mice (31), and the prevalence of chaphamaparvoviruses in mouse liver tissues was high, suggesting that it may also be a gastrointestinal pathogen (9). Although there is no clear disease association, most infected dogs became ill, and one cachavirus-positive dog died. Therefore, the involvement of cachavirus in a small proportion of diarrheic dogs cannot be ruled out.

In this study, we have shown that cachavirus is often involved in coinfections with other viruses. All cachavirus-positive samples were coinfected with one or more other viruses. The coinfection rate was similar to that reported in China in 2018–2019 (17) and in Canada in 2022 (15). In those studies, the coinfection of cachavirus with CPV-2 was most frequent, whereas in our study, its coinfection with CCoV was most frequent (18). We also detected coinfections with CaAstV (18.2%) for the first time. Previous studies have shown that cachavirus easily establishes infections in already-infected hosts (15), suggesting that coinfection with cachavirus is widespread. It should be noted that dogs infected with cachavirus and multiple other viruses showed obvious clinical signs. Only four dogs coinfected with CCoV showed no symptoms, whereas the rest had diarrhea. The coinfecting viruses (CCoV, CaAstV, and CDV) are all clinically associated with severe diarrhea (32–34). Interestingly, both CCoV and cachavirus can be detected in asymptomatic dogs, but most coinfections of these two viruses cause diarrhea. Therefore, we infer that coinfection with other viruses increases the infectivity of cachavirus and aggravates its clinical symptoms. In particular, coinfections of CCoV and cachavirus may increase the pathogenicity of both. In daily life, diarrhea has always been a common disease affecting the health of dogs and can lead to death in severe cases. For a long time, there has been no preventive treatment for diarrhea in dogs, which may be related to complex infections of multiple viruses. Therefore, further study of the degree to which coinfection influences diarrhea in dogs, and the mechanism(s) by which coinfections affect clinical signs is required.

Cachavirus can infect multiple hosts, with potential transmission between them. Cachavirus was first detected in dogs, but was also detected in cats in 2018–2019 (16). In the latest study, cachavirus was found in wild animals, including coyotes (15, 17), and appears to have been circulating among wild animals for at least 10 years. Therefore, cachavirus may spread among wild animals, dogs, and cats, especially because both coyotes and dogs are canids, which may all be susceptible to this virus. Consequently, the hosts and transmission modes of cachavirus warrant intensive research.

With an amino acid analysis, we identified a clear hypervariable region in cachavirus NS1. The region is located at residues 252–255 (Ser252Cys, Gly253Leu, Gly254Thr, and Tyr255Phe), and is consistent with previous Chinese strains (16, 17). Therefore, we suspect that this mutation may be a unique change in the evolution of cachaviruses in China, but its role requires further study. In comparisons of the NS1 and VP1 sequences, strains CY56 and NWT-W78 showed different mutation sites from other strains, consistent with their positions on the phylogenetic trees.

To analyze the synonymous codon usage patterns in these cachavirus strains, we calculated the RSCU of the VP1 genes. To clarify the molecular evolution of individual cachavirus genes, we investigated the use of synonymous codons, to better understand the regulation of viral gene expression (35, 36). In most studies, the main factor causing variations in codon usage in genes with high A + T or G + C contents is mutation bias (37–40). Cachavirus VP1 shows the same preference for A/T-ending codons as the VP gene of CPV. However, the codon GGA (Gly) is preferred in cachavirus, whereas GGT (Gly) is preferred in CPV-2. The codon repertoire of cachavirus does not include GAG (Glu), whereas its RSCU in CPV-2 is 0.414 (41). This is an obvious difference between cachavirus and CPV-2. More comprehensive analyzes are required to determine the true extent of the codon usage bias changes within and among cachavirus strains and the effects of factors such as the dominant host species, transmission mechanisms, cell tropism, and the genetic structure of virus (42).

We conducted the first co-evolutionary analysis of cachavirus and other chaphamaparvoviruses, to understand their development and spread. Cachavirus showed significant cospeciation with Feline chapparvovirus, and the cachavirus has undergone host-switching events from dog to cat. Cats can be infected by both cachavirus and Feline chapparvovirus, although the divergence between the two viruses is nearly 30%, suggesting that they are two distinct species, and have been classified as *Chaphamaparvovirus carnivoran 1 and 2*. We speculate that the emergence of cachavirus in cats is probably the result of host-switching from dogs. In canine cachavirus, multiple cospeciation and host-switching events have occurred among wolves, coyotes, and dogs. From other co-evolutionary and transmission events, it can be inferred that chaphamaparvoviruses may have also been transmitted between wild and domestic animals. Therefore, appropriate measures must be taken to prevent the spread and evolution of cachavirus.

In summary, this is the first report of the presence of cachavirus in dogs in northeastern China. Cachavirus strains often coexist with other canine intestinal pathogens, and CaAstV was detected in all cachavirus-positive samples, with different incidence rates of enteritic symptoms. Our research also suggests that cachavirus continues to evolve in China, and extends our understanding of the epidemiological status of cachavirus in China. A codon usage analysis showed that most of the preferred codons in cachavirus are A- or T-ending codons, as in traditional canine parvovirus. A co-evolutionary analysis showed that cachavirus has undergone cospeciation and transmission among different host species. Large-scale studies are required to confirm the pathogenicity of this virus.

## Data availability statement

The datasets presented in this study can be found in online repositories. The names of the repository/repositories and accession number(s) can be found at: https://www.ncbi.nlm.nih.gov/nuccore; OQ108871-OQ108876.

## Ethics statement

The animal studies were approved by Approval (NEAUEC20210326, 30 March 2021) was obtained from the Institutional Committee of Northeast Agricultural University for animal experiments. The studies were conducted in accordance with the local legislation and institutional requirements. Written informed consent was obtained from the owners for the participation of their animals in this study.

## Author contributions

YB, XY, ZG, KX, ZY, and HS contributed to sample collection and conducted experiments. NL drafted the manuscript and analyzed data. JG and LZ critically revised the manuscript and gave final approval. All authors contributed to the article and approved the submitted version.

## Funding

This research was supported by the National Key R&D Program of China (2021YFF0703000), the SIPT program of Northeast Agricultural University (202210224166 and S202210224168).

## Conflict of interest

The authors declare that the research was conducted in the absence of any commercial or financial relationships that could be construed as a potential conflict of interest.

## Publisher’s note

All claims expressed in this article are solely those of the authors and do not necessarily represent those of their affiliated organizations, or those of the publisher, the editors and the reviewers. Any product that may be evaluated in this article, or claim that may be made by its manufacturer, is not guaranteed or endorsed by the publisher.
