# Supplementary material for: The prevalence, genetic diversity and evolutionary analysis of cachavirus firstly detected in northeastern China
Source: Front Vet Sci. 2023 Sep 13;10:1233972. doi: 10.3389/fvets.2023.1233972 (PMC10527371; doi:10.3389/fvets.2023.1233972)
Supplement: Supplementary file 1 [file Data_Sheet_1.docx]

Supplementary Material

The Prevalence, genetic diversity and evolutionary analysis of Cachavirus firstly detected in northeastern China

Nuowa Li^1,2^, Yue Bai^2^, Xin Yan^3^, Zhiyuan Guo^2^, Kongrui Xiang^2^, Zaixing Yang^2^，Haikun Shangguan^2^, Junwei Ge^2,4*^, Lili Zhao^1*^

*** Correspondence:**

Dr. Lili Zhao

E-mail addresses: zhaolili@jlu.edu.cn (L. Zhao)

Dr. Junwei Ge E-mail addresses: gejunwei@neau.edu.cn (J. Ge)

**Supplementary Figure 1.**


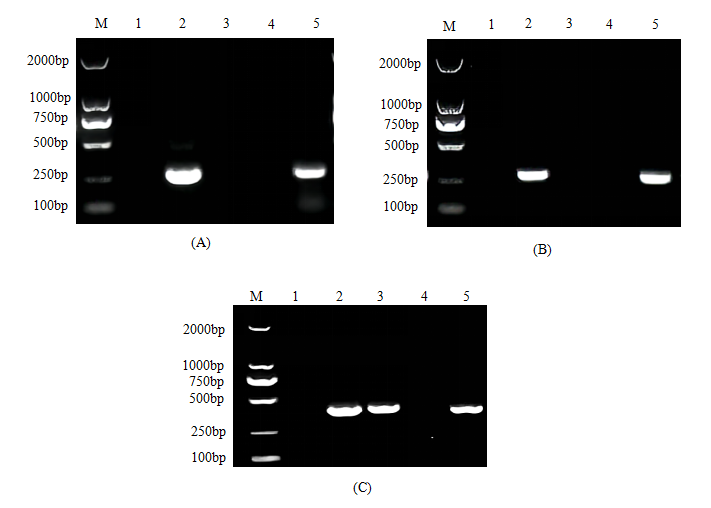


**Supplementary Figure 1.** Coinfection of cachavirus-positive samples was detected with PCR. (A) PCR results for canine distemper virus (CDV). (B) PCR results for canine astrovirus (CaAstV). (C) PCR results for canine coronavirus (CCoV).

**Supplementary Table 1. RSCU analysis of cachavirus VP1 gene.**

| AA | Codon | Cachavirus-1A | Cachavirus-1B | MT123283 | MT123284 | MT123285 | MT123286 | MT123287 | MN928790 | MN928791 | A8 | F10 | K46 |
| --- | --- | --- | --- | --- | --- | --- | --- | --- | --- | --- | --- | --- | --- |
| Phe | TTT | 2.00 | 2.00 | 2.00 | 2.00 | 1.90 | 2.00 | 2.00 | 2.00 | 2.00 | 2.00 | 2.00 | 2.00 |
|  | TTC | 0 | 0 | 0 | 0 | 0.10 | 0 | 0 | 0 | 0 | 0 | 0 | 0 |
| Leu | TTA | 2.73 | 2.87 | 2.73 | 2.73 | 2.73 | 2.73 | 2.73 | 2.73 | 2.73 | 2.35 | 3.00 | 3.00 |
|  | TTG | 0.27 | 0 | 0.27 | 0.27 | 0.27 | 0.27 | 0.27 | 0.27 | 0 | 0.26 | 0 | 0 |
|  | CTT | 0.82 | 1.30 | 1.09 | 0.82 | 1.09 | 1.09 | 1.09 | 1.09 | 1.09 | 1.04 | 1.09 | 1.09 |
|  | CTC | 0.82 | 0.52 | 0.55 | 0.82 | 0.55 | 0.55 | 0.55 | 0.55 | 0.55 | 0.52 | 0.55 | 0.55 |
|  | CTA | 1.36 | 1.30 | 1.36 | 1.36 | 1.36 | 1.36 | 1.09 | 1.36 | 1.64 | 1.83 | 1.36 | 1.36 |
|  | CTG | 0 | 0 | 0 | 0 | 0 | 0 | 0.27 | 0 | 0 | 0 | 0 | 0 |
| Ile | ATT | 1.85 | 1.73 | 1.85 | 1.80 | 1.75 | 1.85 | 1.80 | 1.85 | 1.85 | 1.85 | 1.85 | 1.80 |
|  | ATC | 0.12 | 0.12 | 0.12 | 0.12 | 0.13 | 0.12 | 0.12 | 0.12 | 0.12 | 0.12 | 0.12 | 0.12 |
|  | ATA | 1.04 | 1.15 | 1.04 | 1.08 | 1.13 | 1.04 | 1.08 | 1.04 | 1.04 | 1.04 | 1.04 | 1.08 |
| Met | ATG | 1.00 | 1.00 | 1.00 | 1.00 | 1.00 | 1.00 | 1.00 | 1.00 | 1.00 | 1.00 | 1.00 | 1.00 |
| Val | GTT | 2.00 | 2.22 | 1.78 | 1.89 | 2.00 | 1.78 | 1.78 | 1.78 | 1.56 | 1.78 | 1.78 | 2.11 |
|  | GTC | 0 | 0 | 0.22 | 0.21 | 0.20 | 0.22 | 0.22 | 0.22 | 0.44 | 0.22 | 0.22 | 0 |
|  | GTA | 1.78 | 1.56 | 1.56 | 1.68 | 1.80 | 1.78 | 1.78 | 1.56 | 1.56 | 1.78 | 1.33 | 1.89 |
|  | GTG | 0.22 | 0.22 | 0.44 | 0.21 | 0 | 0.22 | 0.22 | 0.44 | 0.44 | 0.22 | 0.67 | 0 |
| Ser | TCT | 2.06 | 2.06 | 2.06 | 2.06 | 2.06 | 2.06 | 2.17 | 2.06 | 2.06 | 2.06 | 2.06 | 2.06 |
|  | TCC | 0.34 | 0.34 | 0.34 | 0.34 | 0.34 | 0.34 | 0.33 | 0.34 | 0.34 | 0.34 | 0.34 | 0.34 |
|  | TCA | 1.89 | 1.71 | 1.89 | 1.89 | 1.89 | 1.89 | 1.83 | 1.89 | 1.89 | 1.89 | 2.06 | 1.89 |
|  | TCG | 0.17 | 0.34 | 0.17 | 0.17 | 0.17 | 0.17 | 0.17 | 0.17 | 0.17 | 0.17 | 0 | 0.17 |
|  | AGT | 0.51 | 0.51 | 0.51 | 0.51 | 0.69 | 0.51 | 0.50 | 0.51 | 0.51 | 0.51 | 0.51 | 0.51 |
|  | AGC | 1.03 | 1.03 | 1.03 | 1.03 | 0.86 | 1.03 | 1.00 | 1.03 | 1.03 | 1.03 | 1.03 | 1.03 |
| Pro | CCT | 1.38 | 1.38 | 1.63 | 1.58 | 1.58 | 1.50 | 1.50 | 1.50 | 1.50 | 1.45 | 1.38 | 1.38 |
|  | CCC | 0.50 | 0.63 | 0.50 | 0.61 | 0.61 | 0.63 | 0.63 | 0.50 | 0.63 | 0.73 | 0.50 | 0.50 |
|  | CCA | 2.00 | 1.88 | 1.63 | 1.58 | 1.70 | 1.75 | 1.63 | 1.88 | 1.75 | 1.70 | 2.00 | 2.00 |
|  | CCG | 0.13 | 0.13 | 0.25 | 0.24 | 0.12 | 0.13 | 0.25 | 0.13 | 0.13 | 0.12 | 0.13 | 0.13 |
| Thr | ACT | 1.25 | 1.33 | 1.25 | 1.25 | 1.33 | 1.25 | 1.31 | 1.33 | 1.23 | 1.28 | 1.23 | 1.25 |
|  | ACC | 0.31 | 0.39 | 0.39 | 0.39 | 0.39 | 0.39 | 0.38 | 0.39 | 0.46 | 0.32 | 0.46 | 0.39 |
|  | ACA | 1.96 | 1.96 | 1.88 | 1.80 | 1.88 | 1.88 | 1.85 | 1.80 | 1.85 | 1.92 | 1.92 | 1.88 |
|  | ACG | 0.47 | 0.31 | 0.47 | 0.55 | 0.39 | 0.47 | 0.46 | 0.47 | 0.46 | 0.48 | 0.38 | 0.47 |
| Ala | GCT | 1.71 | 1.71 | 1.71 | 1.63 | 1.48 | 1.71 | 1.71 | 1.71 | 1.71 | 1.71 | 1.71 | 1.71 |
|  | GCC | 0.14 | 0.14 | 0.14 | 0.15 | 0.30 | 0.14 | 0.14 | 0.14 | 0.14 | 0.14 | 0.14 | 0.14 |
|  | GCA | 2.14 | 2.14 | 2.14 | 2.22 | 2.22 | 2.14 | 2.14 | 2.14 | 2.14 | 2.14 | 2.14 | 2.14 |
| Tyr | TAT | 1.21 | 1.33 | 1.29 | 1.26 | 1.29 | 1.29 | 1.26 | 1.26 | 1.29 | 1.36 | 1.21 | 1.21 |
|  | TAC | 0.79 | 0.67 | 0.71 | 0.74 | 0.71 | 0.71 | 0.74 | 0.74 | 0.71 | 0.64 | 0.79 | 0.79 |
| His | CAT | 1.82 | 1.82 | 1.82 | 1.82 | 1.82 | 1.82 | 1.82 | 1.83 | 1.82 | 1.82 | 1.82 | 1.82 |
|  | CAC | 0.18 | 0.18 | 0.18 | 0.18 | 0.18 | 0.18 | 0.18 | 0.17 | 0.18 | 0.18 | 0.18 | 0.18 |
| Gln | CAA | 1.71 | 1.71 | 1.71 | 1.71 | 1.71 | 1.57 | 1.71 | 1.69 | 1.71 | 1.69 | 1.86 | 1.71 |
|  | CAG | 0.29 | 0.29 | 0.29 | 0.29 | 0.29 | 0.43 | 0.29 | 0.31 | 0.29 | 0.31 | 0.14 | 0.29 |
| Asn | AAT | 1.53 | 1.49 | 1.53 | 1.47 | 1.53 | 1.53 | 1.54 | 1.53 | 1.58 | 1.53 | 1.58 | 1.53 |
|  | AAC | 0.47 | 0.51 | 0.47 | 0.53 | 0.47 | 0.47 | 0.46 | 0.47 | 0.42 | 0.47 | 0.42 | 0.47 |
| Lys | AAA | 2.00 | 1.83 | 2.00 | 2.00 | 1.92 | 1.91 | 1.84 | 2.00 | 1.92 | 1.91 | 2.00 | 2.00 |
|  | AAG | 0 | 0.17 | 0 | 0 | 0.08 | 0.09 | 0.16 | 0 | 0.08 | 0.09 | 0 | 0 |
| Asp | GAT | 1.52 | 1.52 | 1.52 | 1.52 | 1.52 | 1.52 | 1.50 | 1.52 | 1.52 | 1.52 | 1.52 | 1.52 |
|  | GAC | 0.48 | 0.48 | 0.48 | 0.48 | 0.48 | 0.48 | 0.50 | 0.48 | 0.48 | 0.48 | 0.48 | 0.48 |
| Glu | GAA | 2.00 | 2.00 | 2.00 | 2.00 | 2.00 | 2.00 | 2.00 | 2.00 | 2.00 | 2.00 | 2.00 | 2.00 |
| Cys | TGT | 1.67 | 1.67 | 1.67 | 1.71 | 1.67 | 1.67 | 1.71 | 1.67 | 1.67 | 1.67 | 1.67 | 1.67 |
|  | TGC | 0.33 | 0.33 | 0.33 | 0.29 | 0.33 | 0.33 | 0.29 | 0.33 | 0.33 | 0.33 | 0.33 | 0.33 |
| Trp | TGG | 1.00 | 1.00 | 1.00 | 1.00 | 1.00 | 1.00 | 1.00 | 1.00 | 1.00 | 1.00 | 1.00 | 1.00 |
| Arg | CGT | 0.90 | 0.86 | 1.00 | 0.90 | 0.90 | 0.82 | 0.95 | 0.90 | 0.95 | 0.90 | 0.95 | 0.90 |
|  | CGC | 0.60 | 0.57 | 0.67 | 0.60 | 0.60 | 0.55 | 0.63 | 0.60 | 0.63 | 0.60 | 0.63 | 0.60 |
|  | CGA | 1.50 | 1.43 | 1.67 | 1.50 | 1.50 | 1.36 | 1.58 | 1.80 | 1.58 | 1.50 | 1.58 | 1.50 |
|  | AGA | 2.40 | 2.86 | 2.33 | 2.40 | 2.70 | 3.00 | 2.53 | 2.40 | 2.53 | 2.70 | 2.21 | 2.70 |
|  | AGG | 0.60 | 0.29 | 0.33 | 0.60 | 0.30 | 0.27 | 0.32 | 0.30 | 0.32 | 0.30 | 0.63 | 0.30 |
| Gly | GGT | 1.17 | 1.17 | 0.96 | 1.00 | 0.83 | 1.04 | 1.00 | 1.00 | 0.96 | 1.00 | 0.96 | 1.17 |
|  | GGC | 0.33 | 0.33 | 0.48 | 0.50 | 0.67 | 0.52 | 0.50 | 0.50 | 0.48 | 0.50 | 0.48 | 0.33 |
|  | GGA | 2.50 | 2.50 | 2.56 | 2.50 | 2.50 | 2.43 | 2.33 | 2.33 | 2.56 | 2.50 | 2.56 | 2.50 |
|  | GGG | 0 | 0 | 0 | 0 | 0 | 0 | 0.17 | 0.17 | 0 | 0 | 0 | 0 |

**Supplementary Table 2A.** Comparison of the VP1 nucleotide and amino acid mutations in the strains reported in this study.

| Cachavirus | % Nucleotide sequence identity | | | | | | | | | | | | | | | | | | |
| --- | --- | --- | --- | --- | --- | --- | --- | --- | --- | --- | --- | --- | --- | --- | --- | --- | --- | --- | --- |
|  | MH893926 | MK448316 | MT123283 | MT123284 | MT123285 | MT1223286 | MT123287 | MT710947 | MT710948 | 0K546100 | OK546101 | OK546102 | OM640108 | OM640109 | MN928790 | MN928791 | A8 | F10 | K46 |
| % amino acid sequence identity | | | | | | | | | | | | | | | | | | | |
| MH893826 |  | 99.4 | 99.0 | 99.0 | 98.9 | 98.9 | 99.1 | 99.5 | 99.5 | 99.0 | 99.4 | 93.4 | 98.9 | 93.2 | 98.7 | 99.0 | 99.0 | 98.9 | 99.1 |
| MK448316 | 99.6 |  | 98.6 | 99.0 | 99.1 | 98.6 | 99.1 | 99.1 | 99.1 | 99.2 | 99.5 | 93.2 | 99.1 | 93.0 | 98.7 | 99.0 | 98.9 | 98.9 | 98.9 |
| MT123283 | 99.4 | 98.9 |  | 98.2 | 98.2 | 98.3 | 98.4 | 99.2 | 99.2 | 98.6 | 98.8 | 93.0 | 98.4 | 92.9 | 97.9 | 98.2 | 98.4 | 98.2 | 98.5 |
| MT123284 | 99.4 | 99.4 | 98.7 |  | 98.9 | 98.7 | 99.2 | 98.7 | 98.7 | 98.9 | 99.2 | 92.9 | 98.9 | 92.7 | 98.9 | 99.4 | 98.9 | 99.1 | 98.8 |
| MT123285 | 99.6 | 99.1 | 98.9 | 98.9 |  | 98.9 | 99.2 | 98.7 | 98.7 | 98.9 | 99.3 | 92.7 | 98.9 | 92.5 | 98.7 | 98.9 | 98.8 | 99.0 | 98.4 |
| MT123286 | 99.6 | 99.1 | 98.9 | 98.9 | 99.6 |  | 98.9 | 98.8 | 98.8 | 98.6 | 98.9 | 92.8 | 98.7 | 92.6 | 98.5 | 98.7 | 98.6 | 98.8 | 98.2 |
| MT123287 | 99.4 | 99.4 | 98.7 | 99.1 | 98.9 | 98.9 |  | 98.9 | 98.9 | 99.1 | 99.4 | 93.1 | 99.0 | 92.8 | 99.2 | 99.2 | 99.2 | 99.4 | 98.7 |
| MT710947 | 100.0 | 99.6 | 99.4 | 99.4 | 99.6 | 99.6 | 99.4 |  | 100.0 | 98.9 | 99.2 | 93.6 | 98.9 | 93.3 | 98.4 | 98.7 | 98.8 | 98.7 | 98.9 |
| MT710948 | 100.0 | 99.6 | 99.4 | 99.4 | 99.6 | 99.6 | 99.4 | 100.0 |  | 98.9 | 99.2 | 93.6 | 98.9 | 93.3 | 98.4 | 98.7 | 98.8 | 98.7 | 98.9 |
| OK546100 | 99.8 | 99.8 | 99.1 | 99.6 | 99.4 | 99.4 | 99.6 | 99.8 | 99.8 |  | 99.6 | 93.2 | 99.1 | 93.0 | 98.7 | 98.9 | 98.7 | 98.9 | 98.8 |
| OK546101 | 99.8 | 99.8 | 99.1 | 99.6 | 99.4 | 99.4 | 99.6 | 99.8 | 99.8 | 100.0 |  | 93.3 | 99.4 | 93.1 | 99.0 | 99.2 | 99.1 | 99.3 | 99.0 |
| OK546102 | 99.6 | 99.6 | 98.9 | 99.4 | 99.1 | 99.1 | 99.4 | 99.6 | 99.6 | 99.8 | 99.8 |  | 93.1 | 97.8 | 92.8 | 92.9 | 92.9 | 93.0 | 93.1 |
| OM640108 | 99.1 | 99.1 | 98.5 | 98.9 | 98.7 | 98.7 | 98.9 | 99.1 | 99.1 | 99.4 | 99.4 | 99.6 |  | 92.9 | 98.6 | 98.8 | 98.7 | 98.9 | 98.6 |
| OM640109 | 99.4 | 99.4 | 98.7 | 99.1 | 98.9 | 98.9 | 99.1 | 99.4 | 99.4 | 99.6 | 99.6 | 99.8 | 99.4 |  | 92.5 | 92.7 | 92.6 | 92.6 | 92.8 |
| MN928790 | 98.7 | 98.7 | 98.1 | 98.9 | 98.3 | 98.3 | 98.5 | 98.7 | 98.7 | 98.9 | 98.9 | 98.7 | 98.3 | 98.5 |  | 98.9 | 98.8 | 99.0 | 98.3 |
| MN928791 | 99.1 | 99.1 | 98.5 | 99.4 | 98.7 | 98.7 | 98.9 | 99.1 | 99.1 | 99.4 | 99.4 | 99.1 | 98.7 | 98.9 | 98.7 |  | 98.9 | 99.1 | 98.8 |
| A8 | 99.4 | 99.4 | 98.7 | 99.4 | 98.9 | 98.9 | 99.1 | 99.4 | 99.4 | 99.6 | 99.6 | 99.4 | 98.9 | 99.1 | 98.7 | 99.1 |  | 99.4 | 99.2 |
| F10 | 99.4 | 99.4 | 98.7 | 99.1 | 98.9 | 98.9 | 99.1 | 99.4 | 99.4 | 99.6 | 99.6 | 99.4 | 98.9 | 99.1 | 98.5 | 98.9 | 99.1 |  | 98.6 |
| K46 | 99.4 | 99.4 | 98.7 | 99.4 | 98.9 | 98.9 | 99.1 | 99.4 | 99.4 | 99.6 | 99.6 | 99.4 | 98.9 | 99.1 | 98.7 | 99.1 | 100.0 | 99.1 |  |

| Cachavirus | % Nucleotide sequence identity | | | | | | | | | | | | | | | | | | |
| --- | --- | --- | --- | --- | --- | --- | --- | --- | --- | --- | --- | --- | --- | --- | --- | --- | --- | --- | --- |
|  | MH893926 | MK448316 | MT123283 | MT123284 | MT123285 | MT1223286 | MT123287 | MT710947 | MT710948 | 0K546100 | OK546101 | OK546102 | OM640108 | OM640109 | MN928790 | MN928791 | A8 | F10 | K46 |
| % amino acid sequence identity | | | | | | | | | | | | | | | | | | | |
| MH893826 |  | 100.0 | 98.6 | 98.3 | 98.4 | 98.7 | 98.9 | 99.1 | 98.9 | 98.7 | 98.8 | 92.6 | 98.2 | 93.0 | 98.7 | 98.9 | 98.3 | 98.9 | 98.6 |
| MK448316 | 100.0 |  | 98.6 | 98.3 | 98.4 | 98.7 | 98.9 | 99.1 | 98.9 | 98.7 | 98.8 | 92.6 | 98.2 | 93.0 | 98.7 | 98.9 | 98.3 | 98.9 | 98.6 |
| MT123283 | 98.5 | 98.5 |  | 99.3 | 98.4 | 98.9 | 99.1 | 99.1 | 98.9 | 99.1 | 99.4 | 92.4 | 98.8 | 92.8 | 99.3 | 99.1 | 98.9 | 99.5 | 99.2 |
| MT123284 | 98.2 | 98.2 | 99.1 |  | 98.1 | 98.6 | 99.0 | 98.8 | 98.7 | 98.8 | 99.1 | 92.3 | 98.5 | 92.7 | 99.0 | 98.8 | 98.6 | 99.2 | 98.9 |
| MT123285 | 98.5 | 98.5 | 98.5 | 98.2 |  | 98.7 | 98.7 | 98.5 | 98.3 | 98.5 | 98.8 | 92.1 | 98.2 | 92.9 | 98.7 | 98.7 | 98.3 | 98.5 | 98.6 |
| MT123286 | 98.5 | 98.5 | 98.5 | 98.2 | 99.4 |  | 99.2 | 98.6 | 98.4 | 98.6 | 98.9 | 92.5 | 98.3 | 92.7 | 98.8 | 99.2 | 98.4 | 99.0 | 98.7 |
| MT123287 | 98.5 | 98.5 | 98.5 | 98.8 | 98.8 | 98.8 |  | 98.8 | 98.6 | 98.8 | 99.1 | 92.3 | 98.5 | 92.7 | 99.0 | 99.4 | 98.6 | 99.2 | 98.9 |
| MT710947 | 98.8 | 98.8 | 99.7 | 99.4 | 98.8 | 98.8 | 98.8 |  | 99.8 | 99.4 | 99.5 | 92.9 | 98.8 | 93.3 | 99.2 | 98.8 | 98.8 | 99.4 | 99.1 |
| MT710948 | 98.5 | 98.5 | 99.4 | 99.1 | 98.5 | 98.5 | 98.5 | 99.7 |  | 99.2 | 99.3 | 92.9 | 98.6 | 93.3 | 99.0 | 98.6 | 98.6 | 99.2 | 98.9 |
| OK546100 | 98.5 | 98.5 | 99.4 | 99.1 | 98.5 | 98.5 | 98.5 | 99.7 | 99.4 |  | 99.5 | 92.8 | 98.8 | 93.0 | 99.2 | 98.8 | 99.0 | 99.4 | 99.3 |
| OK546101 | 98.8 | 98.8 | 99.7 | 99.4 | 98.8 | 98.8 | 98.8 | 100.0 | 99.7 | 99.7 |  | 92.6 | 99.1 | 93.0 | 99.5 | 99.1 | 99.1 | 99.5 | 99.4 |
| OK546102 | 97.6 | 97.6 | 98.5 | 98.2 | 97.6 | 97.6 | 97.6 | 98.8 | 98.5 | 98.5 | 98.8 |  | 92.6 | 97.5 | 92.6 | 92.3 | 92.8 | 92.8 | 92.9 |
| OM640108 | 98.2 | 98.2 | 99.1 | 99.1 | 98.2 | 98.2 | 98.5 | 99.4 | 99.1 | 99.1 | 99.4 | 98.2 |  | 93.0 | 98.9 | 98.5 | 98.5 | 99.1 | 98.8 |
| OM640109 | 97.9 | 97.9 | 98.8 | 98.5 | 97.9 | 97.9 | 97.9 | 99.1 | 98.8 | 98.8 | 99.1 | 99.1 | 98.5 |  | 93.0 | 92.7 | 93.0 | 93.2 | 93.1 |
| MN928790 | 98.8 | 98.8 | 99.7 | 99.4 | 98.8 | 98.8 | 98.8 | 100.0 | 99.7 | 99.7 | 100.0 | 98.8 | 99.4 | 99.1 |  | 99.2 | 99.2 | 99.4 | 99.5 |
| MN928791 | 98.8 | 98.8 | 98.8 | 98.5 | 99.1 | 99.1 | 99.1 | 99.1 | 98.8 | 98.8 | 99.1 | 97.9 | 98.5 | 98.2 | 99.1 |  | 98.6 | 99.2 | 98.9 |
| A8 | 98.8 | 98.8 | 99.7 | 99.4 | 98.8 | 98.8 | 98.8 | 100.0 | 99.7 | 99.7 | 100.0 | 98.8 | 99.4 | 99.1 | 100.0 | 99.1 |  | 99.0 | 99.7 |
| F10 | 98.8 | 98.8 | 99.7 | 99.4 | 98.8 | 98.8 | 98.8 | 100.0 | 99.7 | 99.7 | 100.0 | 98.8 | 99.4 | 99.1 | 100.0 | 99.1 | 100.0 |  | 99.3 |
| K46 | 98.8 | 98.8 | 99.7 | 99.4 | 98.8 | 98.8 | 98.8 | 100.0 | 99.7 | 99.7 | 100.0 | 98.8 | 99.4 | 99.1 | 100.0 | 99.1 | 100.0 | 100.0 |  |

**Supplementary Table 2B.** Comparison of the NS1 nucleotide and amino acid mutations in the strains reported in this study.
