# Supplementary material for: The prevalence, genetic diversity and evolutionary analysis of cachavirus firstly detected in northeastern China
Source: Front Vet Sci. 2023 Sep 13;10:1233972. doi: 10.3389/fvets.2023.1233972 (PMC10527371; doi:10.3389/fvets.2023.1233972)
Supplement: Supplementary file 2 [file Data_Sheet_2.docx]

Supplementary Material

The Prevalence, genetic diversity and evolutionary analysis of Cachavirus firstly detected in northeastern China

Nuowa Li^1,2^, Yue Bai^2^, Xin Yan^3^, Zhiyuan Guo^2^, Kongrui Xiang^2^, Zaixing Yang^2^，Haikun Shangguan^2^, Junwei Ge^2,4*^, Lili Zhao^1*^

*** Correspondence:**Dr. Lili Zhao
E-mail addresses: zhaolili@jlu.edu.cn (L. Zhao)

Dr. Junwei Ge E-mail addresses: gejunwei@neau.edu.cn (J. Ge)

>OQ108874 Cachavirus-K46-VP1

ATGGCTGAAGATGTATCTTTTAACAATACTTATATGGCTTACTGGAATAATATGCCGTATGTGTATCCTAATAATACGCTTGCTACTATTGTTGCTGCTGAAACTGGTGGAAATGCAATAAATACTGGATGGCATATCTTACCTACAATGCAATGGCGTCATTTTATTACCCCTAAACAATGGATAACGATGAATATTAACTATGAAGCATACCATGTCAAAGGATACTCTGTAACCGTATATAATCCAGTACCTATGACACAACAATTAGCAATTCAAGGAACAACTGCATTTACTGCATTTAATAATACTATATACACATTAGGTGCACAAGATGATCTATACGAAACTAATTGGTTTAATTGGTGGAAAGATTCTTCTATGAAAGATTTTTCAATTGCCTATAAAGAAGGCATGTTTAAATCTGGAAGCGACGGTACAACTCAAAAACGAACCATTTTACCAATATATTCATGGGAACCACATAACGCAACAACTGTTGACGATCACACATTTTCAGTTGATTTAACATCTAGTGGATCAGCTGTATGGCCCGCTCCTGATTCCAGCAGCAGGCCTCGAAGACCCACTGGATGTTTTTGGGATCCTTTAAATGATCCATCTTCTATAATGGAACTTCGTCCAGGAAAAAATTCAATGACATGGTCATGGAATGTTCATCCAGCTGATGAAAATCGCTGGTTTAATTTTGACCAAATTGCTAAATGGGCACCATATGTACATGATAATCCATTTTTAAAATACAACATGGCTGGAGGTACTGGTTCTTATATTGTTGCTCCACAAGATGACCCATATAAACTTGCAACAGACAGCAATTGTAATTCTGATCTCGCATCTGATAAAAAATGTGATTATACAATTCCTGACTTAAGCTTTTTACCAATTGTTCCCATGAGTTGGTTTTGGCATGAAATAAATAAATCAATTGCAGCATCTGATGTTATGCGAACGTACTCATGGCGTTTTGATGGAACAGAATACGAACAATATAAATATCCACCTACTCAATGTTTTATTAAAGGATTACCTTTATTTGATGATAACAATACACATATACCTACCACAACACAAGGATGTTTTCAAGTGACTCTACATCTTGCTTGCAAAAAAAGAAGGAGCCGATACTACGCACCAACATGGGGACCATGGACTTGGAAAGATATATATGGCATTGGCTCAGATACGAGAATGTATTCCAATTACATTAGATATAGAACAGGTGGAGCAAGACGAACATGGACCAACGTGGACAAAACCAATACAACGCTAAGTCAGCTAACAAAATTTAGAGAAACACCCTTTGGAACAACACCATATTCAGGAAATACAACATCATCAATTACTGCACCAATAAAAGAACTCGTTAACCTAATTAAACGCCATAAAAA

>OQ108875 Cachavirus-A8-VP1

ATGGCTGAAGATGTATCTTTTAACAATACTTATATGGCTTACTGGAATAATATGCCGTATGTGTATCCTAATAATACGCTTGCTACTATTGTTGCTGCTGAAACTGGTGGAAATGCAATAAATACTGGATGGCATATCTTACCTACAATGCAGTGGCGTCATTTTATTACCCCTAAACAATGGATAACGATGAATATTAACTATGAAGCATACCATGTCAAAGGATACTCTGTAACCGTATATAATCCAGTACCTATGACACAACAATTAGCAATTCAAGGAACAACTGCATTTACTGCATTTAATAATACTATATACACATTAGGTGCACAAGATGATCTATACGAAACTAATTGGTTTAATTGGTGGAAAGATTCTTCTATGAAAGATTTTTCAATTGCCTATAAAGAAGGCATGTTTAAATCTGGAAGCGACGGTACAACTCAAAAACGAACCATTTTACCTATATATTCATGGGAACCACATAACGCAACAACTGTCGACGATCACACGTTTTCAGTTGATTTAACATCTAGTGGATCGGCTGTATGGCCCGCTCCTGATTCCAGCAGCAGGCCTCGAAGACCCACTGGATGTTTTTGGGATCCTTTAAATGATCCCTCTTCTATAATGGAACTTCGTCCAGGAAAAAATTCAATGACATGGTCATGGAATGTTCATCCAGCTGATGAAAATCGCTGGTTTAATTTTGACCAAATTGCTAAATGGGCACCATATGTACATGATAATCCATTTTTAAAATACAACATGGCTGGAGGTACTGGTTCTTATATTGTTGCTCCACAAGATGACCCATATAAACTTGCAACAGACAGCAATTGTAATTCTGATCTCGCATCTGATAAAAAATGTGATTATACAATTCCTGACTTAAGCTTTTTACCAATTGTTCCCATGAGTTGGTTTTGGCATGAAATAAATAAGTCAATTGCAGCATCTGATGTTATGCGAACGTACTCATGGCGTTTTGATGGAACAGAATATGAACAATATAAATATCCACCTACTCAATGTTTTATTAAAGGATTACCTCTATTTGATGATAACAATACACATATACCTACCACAACACAAGGATGTTTTCAAGTAACTCTACATCTTGCTTGCAAAAAAAGAAGAAGCCGATACTACGCACCAACATGGGGACCATGGACTTGGAAAGATATATATGGCATTGGCTCAGATACGAGAATGTATTCCAATTACATTAGATATAGAACAGGTGGAGCAAGACGAACATGGACCAACGTGGACAAAACCAATACAACGCTAAGTCAGCTAACAAAATTTAGAGAAACACCCTTTGGAACAACACCATATTCAGGAAATACAACATCATCAATTACTGCACCAATAAAAGAACTCGTTAACCTAATTAAACGCCATAAAAA

>OQ108876 Cachavirus-F10-VP1

ATGGCTGAAGATGTATCTTTTAACAATACTTATATGGCTTACTGGAATAATATGCCGTATGTATATCCTAATAATACGCTTGCTACTATTGTTGCTGCTGAAACTGGTGGAAATGCAATAAATACTGGATGGCATATCTTACCTACAATGCAGTGGCGTCATTTTATTACCCCTAAACAATGGATAACGATGAATATTAACTATGAAGCATACCATGTTAAAGGATACTCTGTAACCGTATATAATCCAGTACCTATGACACAACAATTAGCAATTCAAGGAACAACTGCATTTACTGCATTTAATAATACTATATACACATTGGGTGCACAAGATGATCTATATGAAACTAATTGGTTTAATTGGTGGAAAGATTCTTCTATGAAAGATTTTTCAATTGCCTATAAAGAAGGCATGTTTAAATCTGGAAGCGACGGTACAACTCTAAAACGAACCATTTTACCTATATATTCATGGGAACCACATAACGCAACAACTGTCGACGATCACACGTTTTCAGTTGATTTAACATCTAGTGGATCGGCTGTATGGCCCGCTCCTGATTCCAGCAGCAGGCCTCGAAGACCCACTGGATGTTTTTGGGATCCTTTAAATGATCCCTCTTCTATAATGGAACTTCGTCCAGGAAAAAATTCAATGACATGGTCATGGAATGTTCATCCAGCTGATGAAAATCGCTGGTTTAATTTTGACCAAATTGCTAAATGGGCACCATATGTACATGATAATCCATTTTTAAAATACAACATGGCTGGAGGTACTGGTTCTTATATTGTTGCTCCACAAGATGACCCATATAAACTTGCAACAGACAGCAATTGTAATTCTGATCTCGCATCTGATAAAAAATGTGATTATACAATTCCTGACTTAAGCTTTTTACCAATTGTTCCCATGAGTTGGTTTTGGCATGAAATAAATAAGTCAATTGCAGCATCTGATGTTATGCGAACGTACTCATGGCGTTTTGATGGAACAGAATATGAACAATATAAATATCCACCTACTCAATGTTTTATTAAAGGATTACCTCTATTTGATGATAACAATACACATATACCTACCACAACACAAGGATGTTTTCAAGTAACTCTACATCTTGCTTGCAAAAAAAGAAGAAGCCGATACTACGCACCAACATGGGGACCATGGACTTGGAAAGATATATATGGCATTGGCTCAGATACGAGAATGTATTCCAATTACATTAGATATAGAACAGGTGGAGCAAGACGAACATGGCCCAACGTGGACAAAAACAATACAACGCTAAGTCAGCTAACAAAATTTAGAGAAACACCCTTTGGAACAACACCATATTCAAGAAATACAACATCATCAATTACTGCACCAATAAAAGAACTCGTTAACCTAATTAAACGCCATTAAAA

>OQ108872 Cachavirus-K46-NS1

AATGGAACGTGGCGGACGTTCTATCACCGGAATCAGACGATTCACGTGGTCGGGAGACAACCTCATTTTGGAAAAAGAGGAAAACATTCAACTAGATAAAAACCAGCTAACACATCAACTTCATATTATGAATGCCCAAACATGGCAAGCTGGTGTTCTTTCTATTACTTATCCTAATGGTTCATCTCCTCTATCTGATCCTTTACCTTATGTTAAATGTTTTGCATCCTTACGATCTGTAAAAGCTTGGATCCTTGCAGGTGAATACAATCCTGAAGGAATATTTCATGTACATTCAATGGCACTTACTTTACAACGCTCTGATTCATTTAGACGTAGTATTGATAGAGAATGGTTCATAAAACGTGTTGAATTTTTACAGTCATTTTCTGATAGAGATCCAGTACTTGATGTATTAAAAATGCAAAAATGTCATAAACCTGAATCTCTTATTGCATATATGTGTAAAGAACCAATATGGATCTGTACTTCAGATAAACATTATACAAATATTGTAACCGCAGTATGCTATTATGATTTAGGAGAACGCTTTCGTATTAAACAACAAGAAAAAATTGAACGTGAACGAGCTAACTCTGCTAATATGAATAAAATTGTTGCTGATGTATTAAATGTTATATATGATCACTCATGTAAAACTATAGAAGATTGCATGAAATGTGCGCCTGATATAATGTCTCAATATTTACATAGATCTGGATTTTCTTCTATTGTACAAAATTGCTTAACATTTGTATCTGCTACTGCACATGGATGGTCACTTGAACGAATTGCTTGTAAACACTTTCCACATCCCGATAACATACATAAATGTTTGTTGCATCAagGTTTAGATGTATGTACATTTGATATATCTTTTTTTAAATGGATAACTAAACAAATGTCCAAACATAACACATTAGTTTTATGGGGTCCTAGTAACACTGGCAAATCTGCCTTTATTTCTGGATTTAAACAATGTGTTTCTTGGGGTG

>OQ108873 Cachavirus-A8-NS1

ACGTGGCGGACGTTCTATCACCGGAATCAGACGATTCACGTGGTCGGGAGACAACCTCATTTTGGAAAAAGAGGAAAACATTCAACTAGATAAAAACCAGCTAACACATCAACTTCATATTATGAATGCCCAAACATGGCAAGCTGGTGTTCTTTCTATTACTTATCCTAATGGTTCATCTCCTCTATCTGATCCTTTACCTTATGTTAAATGTTTTGCATCCTTACGATCTGTAAAAGCTTGGATCCTTGCAGGTGAATACAATCCTGAAGGAATATTTCATGTACATTCAATGGCACTTACTTTACAACGCTCTGATTCATTTAGACGTAGTATTGATAGAGAATGGTTCATAAAACGTGTTGAATTTTTACAGTCATTTTCTGATAGAGATCCAGTACTTGATGTATTAAAAATGCAAAAATGTCATAAACCTGAATCTCTTATTGCATATATGTGTAAAGAACCAATATGGATCTGTACTTCAGATAAACATTACACAAATATTGTAACCGCAGTATGCTATTATGATTTAGGAGAACGCTTTCGTATTAAACAACAAGAAAAAATTGAACGTGAACGAGCTAACTCTGCTAATATGAATAAAATTGTTGCTGATGTATTAAATGTTATATATGATCACTCATGTAAAACTATAGAAGATTGCATGAAATGTGCGCCTGATATAATGTCTCAATATTTACATAGATCTGGGTTTTCTTCTATTGTACAAAATTGCTTAACATTTGTATCTGCTACTGCACATGGATGGTCACTTGAACGAATTGCTTGTAAACACTTTCCACATCCCGATAACATACATAAATGTTTATTGCATCAAGGTTTAGATGTATGTACATTTGATATATCTTTTTTTAAATGGATAACTAAACAAATGTCCAAACATAACACATTAGTTTTATGGGGTCCTAGTAACACTGGCAAATCTGCCTTTATTTCTGGATTTAAACAATGTGTTTCTTGGGGT

>OQ108871 Cachavirus-F10-NS1

GAGATGGAACGTGGCGGACGTTCTATCACCGGAATCAGACGATTCACGTGGTCGGGAGACAACCTCATTTTGGAAAAAGAGGAAAACATTCAACTAGATAAAAACCAGCTAACACATCAACTTCATATTATGAATGCCCAAACATGGCAAGCTGGTGTTCTTTCTATTACTTATCCTAATGGTTCATCTCCTCTATCTGATCCTTTACCTTATGTTAAATGTTTTGCATCCTTACGATCTGTAAAAGCTTGGATCCTTGCAGGTGAATACAATCCTGAAGGAATATTTCATGTACATTCAATGGCACTTACTTTACAACGCTCTGATTCATTTAGACGTAGTATTGATAGAGAATGGTTCATAAAACGTGTTGAATTTTTACAGTCATTTTCTGATAGAGATCCAGTACTTGATGTATTGAAAATGCAAAAATGTCACAAACCTGAATCTCTTATTGCATATATGTGTAAAGAACCAATATGGATCTGTACTTCAGATAAACATTATACAAATATTGTAACCGCAGTATGCTATTATGATTTAGGAGAACGCTTTCGTATTAAACAACAAGAAAAAATTGAACGTGAACGAGCTAACTCTGCTAATATGAATAAAATTGTTGCTGATGTATTGAATGTTATATATGATCACTCATGTAAAACTATAGAAGATTGCATGAAATGTGCGCCTGATATAATGTCTCAATATTTACATAGATCTGGATTTTCTTCTATTGTACAAAACTGCTTAACATTTGTATCTGCTACTGCACATGGATGGTCACTTGAACGAATTGCTTGTAAACACTTTCCACATCCTGATAACATACATAAATGTTTGTTACATCAAGGTTTAGATGTATGTACATTTGATATATCTTTTTTTAAATGGATAACTAAACAAATGTCCAAACATAACACATTAGTTTTATGGGGTCCTAGTAACACTGGCAAATCTGCATTTATTTCTGGATTTAAACAATGTGTTTCTTGGGGTGAAATTGTGATAC
